# Cerebral cryptococcosis with pseudo‐meningiomatous revelation?

**DOI:** 10.1002/ccr3.3441

**Published:** 2020-10-27

**Authors:** Asmaa Hazim, Omar Amriss

**Affiliations:** ^1^ Department of Neurology Faculty of Medicine Cheikh Khalifa Hospital Mohamed VI University of Health Science Casablanca Morocco; ^2^ Department of Radiology Faculty of Medicine Hassan II University 20 Août Hospital Ibn Rochd National Teaching Hospital Casablanca Morocco

**Keywords:** Neurology, Neuromeningeal cryptococcosis, Meningitis, Pseudo‐meningioma

## Abstract

We report the case of a 28‐year‐old woman with history of lupus treated by Mycophenolate Mofetil and admitted for febrile meningeal syndrome associated with diplopia. Both initial and control brain MRI showed pseudo‐meningiomatous lesion while second CSF microscopy revealed characterizing images of Cryptococcus neoformans.

## Graphical abstract

Our case report emphasizes that there is a rare case of cryptococcal meningitis presenting with meningioma‐like lesion especially in the immunocompromised population.

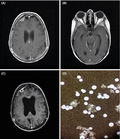

## 1 CASE

A 28‐year‐old woman with history of lupus treated by Mycophenolate Mofetil was admitted for febrile meningeal syndrome associated with diplopia.

**Figure 1:**
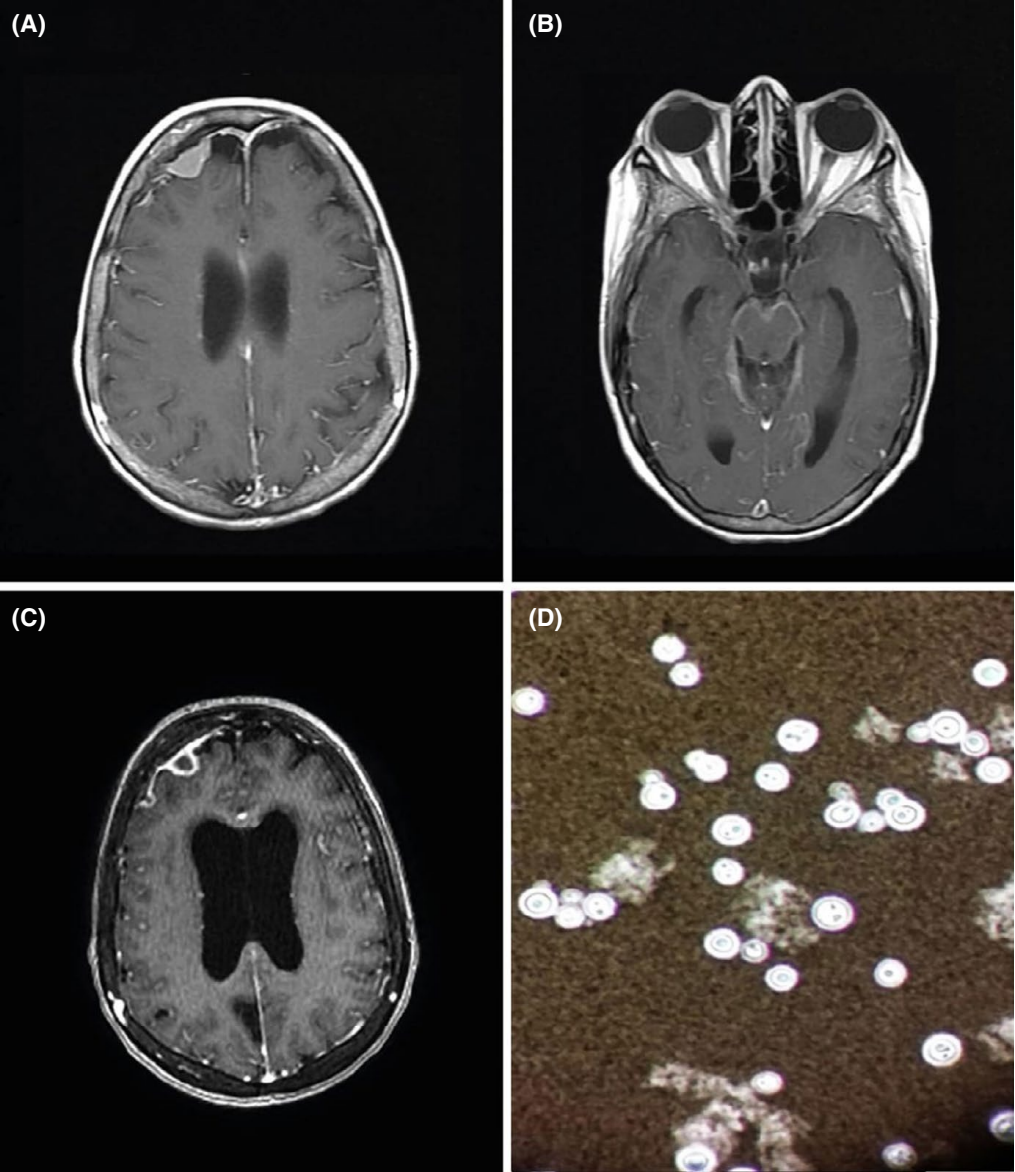
A and B, Initial Brain MRI showing right frontal pseudo‐meningiomatous extra‐axial lesion homogeneously enhanced (A) with diffuse meningeal contrast enhancement (B). C, Second Brain MRI highlighting annular lesions with peripheral enhancement associated with a hydrocephalus. D, CSF microscopy with Indian ink preparation showing encapsulated yeasts with retracted clear halo characterizing *Cryptococcus neoformans*

Brain MRI showed a right frontal pseudo‐meningiomatous extra‐axial lesion, homogeneously enhanced with a diffuse meningeal contrast enhancement after Gadolinium administration. (Figure 1A‐B).

Cerebrospinal fluid (CSF) microscopy performed without Indian ink preparation revealed a lymphocytic meningitis with hyperproteinorachia (2.74 g/L), hypoglycorrhachia (0.19 g/L) but no organisms were seen. CSF culture was sterile and polymerase chain reaction for *Mycobacterium tuberculosis* returned negative. A treatment consisting of a combination of ceftriaxone (6g/day) and aciclovir (10 mg/kg every 8 hours) was initiated.

One week later, the patient's status worsened with deterioration of consciousness associated with generalized seizures of frontal lobe onset. Second Brain MRI highlighted a right frontal extra‐axial annular lesion with peripheral enhancement associated with hydrocephalus (Figure 1C). CSF microscopy was repeated with Indian ink preparation and identified encapsulated yeasts surrounded by retracted clear halo (Figure 1D) characterizing *Cryptococcus neoformans*.^1^, ^2^

After 10 days of fluconazole (400 mg/day)^3^, the patient's clinical status improved, CSF protein decreased to 1 g/L, and CSF glucose increased to 0.34 g/L. However, brain MRI was not repeated after antifungal treatment because the patient was discharged against medical advice.

## CONFLICT OF INTEREST

None declared.

## AUTHOR CONTRIBUTIONS

AH: Design and conceptualization, data collection and analysis, wrote and drafted the manuscript. OA: analyzed the data and revised the manuscript.

## Data Availability

Authors can confirm that all relevant data are included in the article and/or its supplementary information files.
